# Next-Generation Sequencing Panel Test in Myeloid Neoplasms and Evaluation with the Clinical Results

**DOI:** 10.5152/eurasianjmed.2022.21102

**Published:** 2022-06-01

**Authors:** Cigdem Yuce Kahraman, Gulden Sincan, Abdulgani Tatar

**Affiliations:** 1 Department of Medical Genetics, Atatürk University Faculty of Medicine, Erzurum, Turkey; 2 Department of Haematology, Atatürk University Faculty of Medicine, Erzurum, Turkey

**Keywords:** Myeloid, malignancies, NGS, panel testing, myeloid panel testing

## Abstract

**Objective**: Myeloid malignancies are heterogeneous disorders due to defective hematopoiesis and myeloid differentiation of hematopoietic stem/progenitor cell. The molecular landscape of the diseases is complex. Molecular alterations are used for classification and evaluation of prognosis and treatment. We aimed to evaluate the advantages of the next-generation sequencing panel testing in myeloid malignancies and clinical outcomes.

**Materials and Methods**: We evaluated the results of 54 patients who underwent next-generation sequencing myeloid panel testing, with fluorescent in situ hybridization (FISH), polymerase chain reaction results and the clinical outcomes. Target genes in the panel were* ASXL1, CALR, CBL, CEBPA, CSF3R, DNMT3A, EZH2, FLT3, IDH1, IDH2, JAK2, KIT, KRAS, MPL, NPM1, NRAS, RUNX1, SETBP1, SF3B1, SH2B3, SRSF2, TET2, TP53, U2AF1,* and *ZRSR2*.

**Results:** Diagnoses were acute myeloid leukemia, essential thrombocytosis, polistemia vera, primary myelofibrosis, hypereosinophilic syndrome (HES), chronic myeloid leukemia, myelodysplastic syndromes, chronic myelomonocytic leukemia. Twenty-eight missense, 8 frameshift, 5 stop gain, and 3 in-frame mutations were detected. A double mutation was detected in *JAK-2* with next-generation sequencing in the patient who was given a false negative result due to polymerase chain reaction limitation.

**Conclusion**: Screening multiple mutations simultaneously, is time and cost-effective. With the panel test, it is possible to determine the diagnosis, prognosis and targeted treatment options with a single test. Next-generation sequencing myeloid panel tests might be a powerful guide for clinicians.

**Main Points**

- Myeloid malignancies are heterogeneous diseases and the molecular landscape is complicated.
- Due to complex mutations and transformation of cells, screening of multiple genes is useful.
- Next-generation sequencing based technology allows time and cost effective results of multiple genes at once.
- Next-generation sequencing myeloid panel tests provide molecular biomarkers for diagnosis, prognosis, and targeted therapy.

## Introduction

Myeloid malignancies consist of a heterogeneous group of disorders due to defective hematopoiesis of hematopoietic stem/progenitor cell and myeloid differentiation. Myeloproliferative neoplasms (MPN), myelodysplastic syndromes (MDS), and acute myeloid leukemia (AML) are the main disorders in these groups.^1^

Genetic abnormalities are detected in 50-60% of the patients with acute myeloid leukemia by conventional cytogenetic and FISH method.^2^ Cytogenetic abnormalities alone are not sufficient for AML formation, mutations such as *FLT3* and *RAS* must be acquired additionally. Since cytogenetic abnormality was not detected in approximately half of AML cases, pathogenesis is explained by gene mutations.^3^ Mutations classified as class 1 mutations; *FLT3, KRAS, NRAS, CKİT,* and *JAK2* (signaling and kinase pathway), cause increased tyrosine kinase activity, triggering proliferation of the cell. mutations; class 2 mutations; *CEBPA, NPM1* (transcription factors), and *MLL*, prevent apoptosis of the cell and disrupt its differentiation.^4^ In addition, epigenetic mutations such as *TET, ASXL1, IDH1, IDH2*, *EZH2*, *DNMT3,* and RNA splicesome mutations such as* U2AF1, SRSF2, ZRSR2, SF3B1,* and tumor suppressors such as* WT1* and* TP53* have been blamed in the pathogenesis of AML.^5^ Age, performance status of the patient, comorbid diseases, MDS, and myeloproliferative disease history as well as genetic characteristics are determinant in the prognosis of AML. In 2017, the genetic risk assessment of AML was updated by European Leukemia Network and classified as favorable, intermediate, and adverse groups.^6^ Biallelic CEBPA mutation and *NPM1* mutation without *FLT3-ITD* or with *FLT3-ITD* ^low^ are the genetic variations classified in the favorable group.

*NPM1* mutation *FLT3-ITD^high^* and wild-type *NPM1* without *FLT3-ITD* or with *FLT3-ITD^low^* are classified in the intermediate group. Wild-type *NPM1* and *FLT3-ITD^high^*, *RUNX1* mutation, *ASXL1* mutation, and *TP53* mutation are classified in the adverse group.^6^

Screening of *FLT3*, *NPM1*, *CEBPA*, and *KIT* mutations is recommended also by National Comprehensive Cancer Network.^1^ *SF3B1* and actionable* IDH1, IDH2* mutations are also advised to be tested. Common somatic mutations in AML are in *FLT3, NPM1A, DNMT3A* at a rate of 25-30% and IDH1/2, TET2 at a rate of 5-15%. Screening of these mutations is useful for diagnostic, prognostic, and treatment options.^1,7^ Targeted therapy in recent years has led to an improvement in treatment. If a germline mutation is detected in the evaluation of the patient, treatment options can be changed. Individuals at risk in the family should be examined especially before stem cell transplantation.^7^

Myelodysplastic syndrome is a clonal stem cell disease characterized by dysplasia and peripheral blood cytopenias. The main somatic mutations in MDS are *TET2, ASXL1, SRSF2, RUNX1, TP53, EZH2, ZRSR2, SF3B1*, and *ETV6* mutations.^8^ *TP53* mutation is associated with short survival, high risk of AML transformation, increased blast percentage, and complex karyotype.^9^ *SF3B1, SRSF2,* and* U2AF1*, splicesome group gene mutations, are seen in more than 50% of MDS patients. *SF3B1* mutation is considered to be a good prognostic marker in MDS and is associated with the ring-sideroblast (RS) phenotype.* SF3B1* mutation is seen in 60-90% of the patients with RS.^10^ RAS mutation occurs in approximately 10-15% of cases and increases the risk of conversion to AML. *FLT3* mutation is seen in 5% of the cases and is associated with a poor prognosis. *TET2, ASXL1*, and *DNMT3A* mutations are detected in 60-70% of MDS patients. *ASXL1* mutation is known to be a poor prognostic marker.^11^

Chronic myeloproliferative diseases were classified into essential thrombocytosis (ET), primary myelofibrosis (PM), chronic neutrophilic leukemia, chronic eosinophilic leukemia, chronic myeloid leukemia (CML), and unclassified myeloproliferative neoplasia by WHO in 2016.^12^ *JAK2, CALR,* and* MPL* are driver mutations. *JAK2* V617F mutation was detected in 95% of polistemia vera (PV) cases, 55% of ET cases, and 60% of PM cases. *JAK2* exon 12 mutation is seen in approximately 3% of PV cases and these patients are younger and isolated erythrocytosis is more common. Disease outcomes are similar to those of *JAK2* V617F-positive patients.* MPL* mutation is observed in 3% of ET cases and 7% of PM cases and in the presence of MPL mutations, older age, higher platelet count and erythropoietin levels, lower hemoglobin, and bone marrow cellularity are seen. *CALR* mutation is seen in 15-24% of ET and 25-35% of PM cases. *TET2, EZH2, DNMT3A, ASXL1, CBL, IKZF1, IDH1/2, SH2B3, FLT3, SOCS, HMGA2, TP53,* and* NRAS/KRAS* mutations are considered important for prognosis.^13,14^ *ASXL1* and *EZH2* mutations are associated with poor prognosis in PM.^15^ *TET2* and *IDH1/2* mutations facilitate leukemic transformation.

While so many mutations were identified in the myeloid malignancy diseases, it seems reasonable to screen them at once with the panel test. And also it is important to perform multigenic NGS panel tests at different times for clinical follow-up of the patients due to the risk of emergence of new somatic mutations.

## Materials and Methods

We evaluated the results of our 54 patients who had myeloid panel testing due to myeloid malignancy in the last 1 year, retrospectively. This study was approved by Ataturk University Medical Faculty Clinical Research Ethics Committee (B.30.2.ATA.0.01.00/521). Written informed consent was obtained from all participants who participated in this study.

In our study, the QIAact Myeloid DNA UMI Panel was studied with the GeneReader NGS System. Target genes in the panel were* ASXL1, CALR, CBL, CEBPA, CSF3R, DNMT3A, EZH2, FLT3, IDH1, IDH2, JAK2, KIT, KRAS, MPL, NPM1, NRAS, RUNX1, SETBP1, SF3B1, SH2B3, SRSF2, TET2, TP53, U2AF1,* and* ZRSR2*. GeneRead DNA Library Q Kit and the GeneRead Clonal Amp Q Kit were used for target enrichment, library preparation, and clonal amplification. DNA quality assessment was done with the QIAxcel instrument. GeneRead Sequencing Q Kit was used to sequence the amplicon libraries on the GeneReader system. Variants were evaluated with Qiagen Clinical Insight Software which includes Clinvar, CADD, PolyPhen, SIFT, Mutation Taster, BLOSUM, PhyloP, MaxEntSan, Gene Splicer, B-SIFT, HGMD, COSMIC in silico tools.

In addition, FISH and polymerase chain reaction (PCR) test results and clinical history were evaluated.

## Results

Myeloid panel results of the 58 patients evaluated; 3 were children, and 1 was diagnosed with biphenotypic acute leukemia hence they were excluded from the study. The remaining 54 patients were classified as 11 AML, 22 ET, 12 PV, 5 PM, 1 HES, 1 CML, 1 MDS, and 1 chronic myelomonocytic leukemia (CMML) diagnosis. The patients consist of 30 males and 24 females (M/F: 55.6%/44.4%). The mean age of the patients was 43.5 (min: 19 max:74 standard deviation: 15.75); 83% of PV were male and 64% of ET were female. FISH, PCR panel, and NGS results within the scope of indication and clinical follow-up of all patients were also evaluated.

Mutations were detected in 24 of 54 patients by NGS myeloid panel testing. A total of 44 mutations were detected in 19 genes. Mutations include 28 missense, 8 frameshift, 5 stop gain, and 3 in-frame mutations. There were a total of 23 mutations in the AML patients, 7 mutations in the ET patients, 3 mutations in the PM patients, 4 mutations in the PV patients, and 2 mutations in the MDS patients. Mutations evaluated as 8 likely pathogenic and 36 pathogenic mutations according to American College of Medical Genetics and Genomics criteria.^16^

In AML; *FLT3* (4 times), *DNMT3A (3), NPM1 (3)*, *IDH2 (2), NRAS (2), TP53 (2), ASLX1 (2)*, *IDH1 (1), EZH2 (1), TET2 (1), KRAS (1),* and* SF3B1 (1)* mutations were detected. *CALR* (4), *MPL (2)*, and *JAK2 (1)* mutations were found in ET. *JAK2 (2)*, *FLT3 (2)*, *DNMT3A (1)*, and *MPL (1)* mutations in PV and *JAK2( 1)*, *ASLX1 (1)*, and *SRSF2 (1)* mutations in PM, *SETB1 (1)* mutation in MDS were detected.

Six of our patients with AML died. No mutation was detected in one of these cases. Two other AML cases had *FLT3, IDH1, EZH2, IDH2, NRAS, NPM1, DNMT3A, KRAS,* and* SF3B1* mutations. The sole MDS case with *SRSF2, SETB1* mutations, and p53 deletion died.

**Figure 1.**
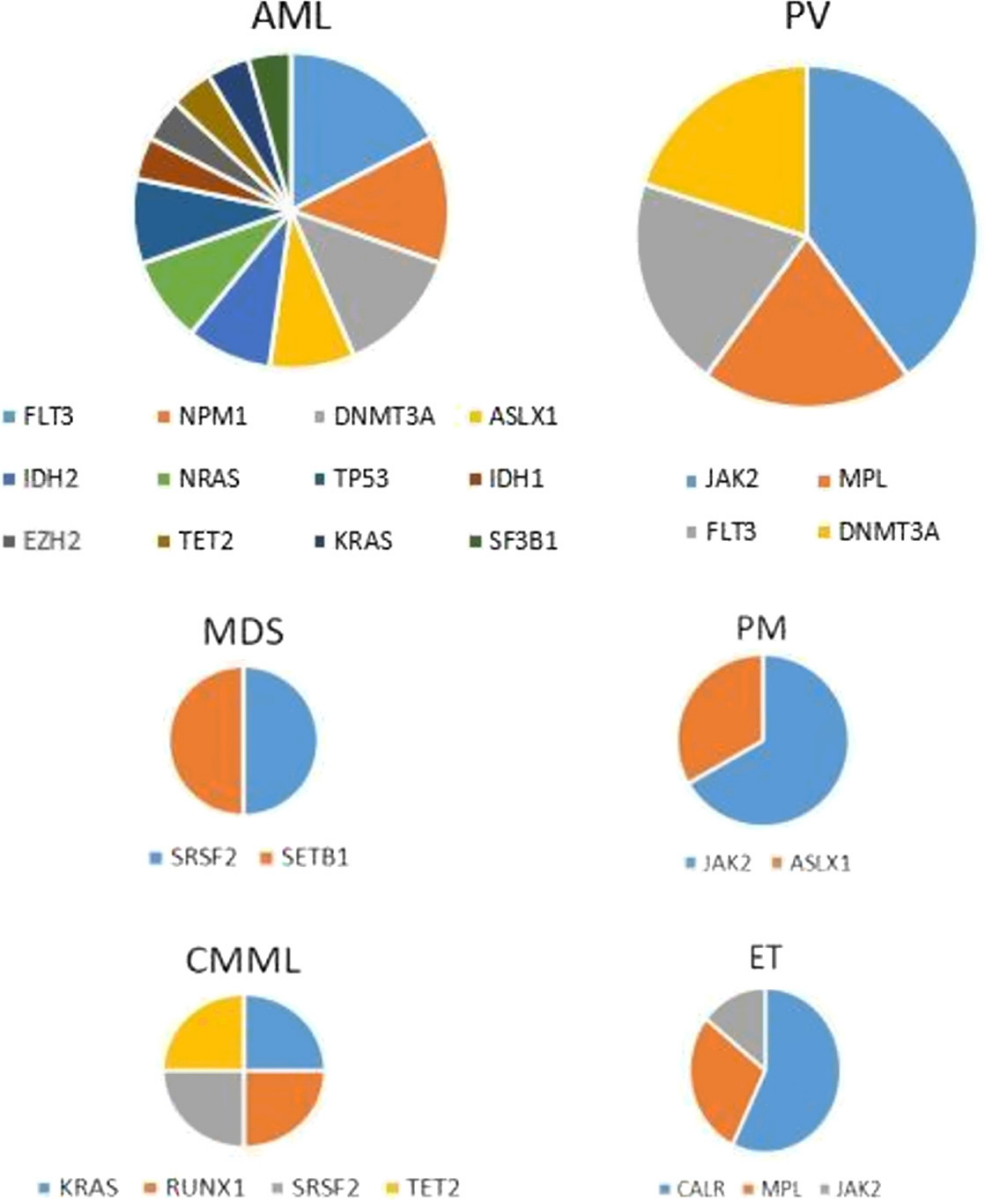
Mutations determined by NGS myeloid panel test according to the diagnosis. AML, acute myeloid leukemia; ET, essential thrombocytosis; PV, polistemia vera; PM, primary myelofibrosis; MDS, myelodysplastic syndrome; CMML, chronic myelomonocytic leukemia.

In the MPN group, 4 of 22 ET patients had *CALR,* 1 had *JAK2 V617F,* and 2 had *MPL* mutation. In 12 patients with PV, 1 had *JAK2 V617F* and *JAK2 C618R* co-mutation, 1 had *FLT3*, 1 had *MPL,* and 1 patient had *DNMT3A* mutations. One of the PM patients had *JAK2 V617F* and the other 1 had *JAK2V617F* and *ASLX1* mutations. There was no mutation in the sole CML and sole HES patient. The CMML patient had *KRAS, RUNX1, SRSF2*, and *TET2* mutations (Figure 1)

We added Tier 1 evident drug recommendations to our reports for 6 patients with *FLT3, IDH1,* and* IDH2* mutations. Recommended therapies:

- *FLT3*-sensitive target therapy; midostaurin, gilteritinib for P3, P4, P16, P37,
- *IDH2*-sensitive target therapy; enasidenib, 5-azacytidine, decitabine for P21
- *IDH1*-sensitive target therapy; ivosidenib, 5-azacytidine, decitabine for P27.

## Discussion

Myeloid malignities are heterogeneous diseases and the molecular landscape is complicated. Next-generation sequencing-based technology allows higher sensitivity than classic molecular tests. Mutations are biomarkers for minimal residual disease, treatment response, and predicting relapse of the disease. Cytogenetics, molecular cytogenetics (FISH), and molecular genetics provide important diagnostic markers for myeloid malignancies. With the improvement of NGS panels, analysis of target mutations in multiple genes become easier and time and cost-effective. Molecular alterations are used for both classification and evaluation of prognosis and treatment.

In AML, there are the United States Food and Drug Administration-approved targeted therapies; for *FLT3* mutations midostaurin and gilteritinib, for *IDH1/2* mutations ivosidenib and enasidenib are the choices. Although we reported the recommended target therapies for 6 of the patients with *FLT3, IDH1,* and *IDH2* mutations. Not *IDH1/2* but *FLT3* targeted therapy just have approved in our country. One of the AML patients was treated with *FLT3-*targeted therapy.

Six of 10 AML patients died and 3 of them had poor prognostic factors; *FLT3*, concordant with the literature. One had *KRAS* and *SF3B1*, 1 had *IDH1* and *EZH2*, 1 had *FLT3* and *NPM1*, and 2 had only *FLT3* mutations. *NRAS/KRAS* mutations, on their own, are not considered to have prognostic significance in antileukemia therapies; however, with *RAS* mutations, the results of high-dose cytarabine remission therapy are better.^17^ Although *NPM1* and *FLT3* co-mutations are classified as the intermediate group,^6^ our 2 patients with these mutations died.

Five of the AML patients are in remission. One of them had *DNMT3A, NRAS, IDH2, ASLX,* and *TP53* mutations, and 1 had *DNMT3A, NRAS, NPM1,* and *IDH2* mutations. It is reported that when *NRAS* mutations are seen together with *NPM1* and *DNMT3A*, it is a moderate prognosis indicator.^18^ Other patient in remission had *RUNX1* mutation, afterwards, it became negative and later had *DNMT3A* mutation. Although the effect of *DNMT3A* and *RUNX1* mutations on prognosis is generally poor, our patient is in remission after treatment. And the other patient had *TET2* mutation which is considered a poor prognostic factor^19^ and is in remission. *IDH1/2* mutations are commonly seen with the co-existence of *NPM1* and *DNMT3A*. Prognostic effect of *IDH1/2* is conflicted.^20^ The epigenetic basis of myeloid malignancies such as classification due to DNA methylation profile is a new issue. *DNMT3A, ASLX,* and* TET2* mutations tend to occur in advanced ages and are associated with poor prognosis. Co-existence with other mutations could change clinical outcomes. Hypomethylating agents are therapy choices for these mutations but it is not approved yet. *TP53* mutations are seen with complex genetic abnormalities.^1^

In MPN patients, mutations other than driver mutations (*JAK2, CALR,* and* MPL)* provide information about prognosis. It was thought that the frequency of *JAK2* mutations in our MPN patients was lower than the rates stated in the literature because the patients who requested a myeloid panel were atypical. A myeloid panel was not requested in classical patients who had already had JAK2 mutation with PCR. One of our PM patients had *JAK2* V617F and *ASLX1* mutations and the other had only *JAK2* V617F mutation. Both of them were in a stable clinical state. It is reviewed that *ASXL1, EZH2, IDH1, IDH2, TP53,* or *SRSF2* mutations are associated with a short survey and leukemic transformation and homozygote *JAK2* V617F mutation causes more clinical symptoms and indicates poor prognosis in PM cases.^21^ Four of the PV patients had mutations; 1 had *JAK2 V617F* ve *JAK2 C618R*, 1 had *DNMT3*, 1 had *MPL,* and the other had *FLT3* mutations. The *FLT3* mutation occurred with an allelic fraction of 50% hence it was confirmed with a mouth swab sample testing if it was a germline mutation. Also, family members at risk are recommended to screen. To the best of our knowledge, co-mutations *JAK2 V617F* ve *JAK2 C618R* are rare and reviewed in 4 studies.^22-25^ In the last study, 2 of 8 polistemia patients were revealed as *JAK2* negative in PCR testing, and later, NGS of *JAK2* coding regions revealed 2 point mutations in exon 14 (c.1849_1853GTCTG>TTTCT; p.V617F/C618L). These mutations in the adjacent codons(617-618) blocked specific primer binding to the target hence a false-negative result occurred. The sanger sequencing of the entire exon 14 or sequencing of the *JAK2* gene entirely by NGS contributes accurate results and diagnosis and it seems like it is not sufficient to scan *JAK2 V617F* with only a PCR test.^25^ In our case *JAK2* V617F mutation was negative in PCR test similarly. Myeloid panel test was studied in this case and a co-mutant genotype was revealed.

Seven of our ET patients had mutations. Four of them had *CALR*, 2 had *MPL*, and 1 had *JAK2 V617F* mutations. Three of 4 *CALR* mutations are type 1 mutations and 1 of them is an uncommon variant. *CALR* mutations in ET consist of type 1 (p.L367fs*46) mutations at a rate of 50% and type 2 (K385fs*47) mutations at a rate of 30%. The prognosis differs according to being type 1 or 2. Type1, but not type 2, is better than *JAK2* mutations.^26^ Common mutations in *MPL,* W515L and W515K and *MPL* mutations, contribute to secondary myelofibrosis and AML.^27^ Two patients had the common variant,W515L and clinic state is stable.

The MDS patient had *SETB1* and poor prognostic marker; *SRSF2* mutations. FISH analysis revealed P53 deletion. The patient did not survive.

Common mutations in CMML are *TET2* (50-60%), *SRSF2* (40-50%), and *ASXL1* (30-40%). *TET2* and *SRSF2* mutations are often seen together. *ASXL1* mutations indicate poor prognosis.^28^ Our CMML patient had *KRAS, RUNX1, TET2,* and* SRSF2* mutations.

In our study both FISH and conventional molecular analysis, if available, were evaluated. There was a concordance in the NGS panel test and reverse transcription-polymerase chain reaction results except for *JAK2 V617F* and *JAK2 C618R* co-mutant patient. A double mutation was detected in *JAK-2* with NGS in the patient who was given a false negative result due to PCR limitation. In addition, we detect various mutations that could be biomarkers for diagnosis, prognosis, and targeted therapies by panel tests.

If we look at the limited aspects of our study, although the total number of patients we evaluated seems sufficient, our sample number is limited when divided into subgroups according to the diagnoses. With more sample numbers, clearer comparisons can be made between mutations and specific diagnoses.

In conclusion, evaluating more biomarkers in 1 step for an accurate diagnosis is valuable for the patients. Multiple mutations are detected in the patients with a single test. Screening multiple mutations at once, saves time and money. Routine clinical usage of NGS panel tests become prevalent in recent years and might be a strong guide for clinicians.
